# Supplementary material for: Physical intimate partner violence and prenatal oral health experiences in the United States
Source: BMC Oral Health. 2023 Oct 12;23:749. doi: 10.1186/s12903-023-03491-0 (PMC10568803; doi:10.1186/s12903-023-03491-0)
Supplement: Supplementary file 1 — Additional file 1: Appendix A. List of Sites and Years in Analytic Sample. Appendix B. Summary Statistics from Pregnancy Risk Assessment Monitoring System, 2016-2019 Stratified by Physical IPV [file 12903_2023_3491_MOESM1_ESM.docx]

**Appendix A: List of Sites and Years in Analytic Sample**

| **State** | **Years** |
| --- | --- |
| Arkansas | 2016, 2018-2019 |
| Colorado | 2016-2019 |
| Connecticut | 2016-2019 |
| Delaware | 2016-2019 |
| Georgia | 2017-2019 |
| Hawaii | 2016, 2019 |
| Iowa | 2016-2019 |
| Indiana | 2018 |
| Kentucky | 2017-2019 |
| Massachusetts | 2016-2019 |
| Maine | 2016-2019 |
| Minnesota | 2018-2019 |
| Missouri | 2016-2019 |
| Mississippi | 2018-2019 |
| Montana | 2017-2019 |
| North Caronia | 2017-2019 |
| North Dakota | 2017-2019 |
| Nebraska | 2016, 2017-2019 |
| New Hampshire | 2016-2019 |
| New Jersey | 2016-2019 |
| New York | 2016-2019 |
| New York City | 2016-2019 |
| Pennsylvania | 2016-2019 |
| Puerto Rico | 2017-2019 |
| Rhode Island | 2016-2019 |
| Tennessee | 2019 |
| Utah | 2016-2019 |
| Virginia | 2016-2019 |
| Washington, DC | 2018-2019 |
| Wisconsin | 2016-2019 |
| West Virginia | 2016-2018 |

**Appendix B: Summary Statistics from Pregnancy Risk Assessment Monitoring System, 2016-2019 Stratified by Physical IPV**

|  | **No IPV**  **(n = 83,583)** | **IPV**  **(n = 1,706)** |  |
| --- | --- | --- | --- |
| **Variables** | **%/ Mean**  **(SD)** | **%/ Mean**  **(SD)** | ***p-value*** |
| *Oral Health Experiences* |  |  |  |
| Didn’t Know Important to Care for Teeth | 11.8% | 19.3% | <.001 |
| Didn’t Talk about Dental Health with Provider | 47.3% | 61.0% | <.001 |
| No Dental Prophylaxis | 51.3% | 63.5% | <.001 |
| Needed to see Dentist for a Problem | 17.9% | 36.0% | <.001 |
| Visited Dentist for Problem | 13.6% | 19.3% | <.001 |
| Visited a Dentist \| Needing to see a Dentist | 68.7% | 57.5% | <.001 |
| *Maternal Age* |  |  |  |
| <24 | 20.0% | 39.5% | <.001 |
| 25-29 | 29.1% | 31.7% | .182 |
| 30-34 | 31.2% | 17.8% | <.001 |
| 35+ | 19.8% | 11.0% | <.001 |
| *Maternal Race/Ethnicity* |  |  |  |
| White | 63.2% | 52.5% | <.001 |
| Hispanic | 15.0% | 15.1% | .954 |
| Black | 13.1% | 23.9% | <.001 |
| Other Race/Ethnicity | 8.6% | 8.4% | .859 |
| *Maternal Educational Attainment* |  |  |  |
| Less than High School | 9.1% | 14.6% | <.001 |
| High School Graduate | 22.5% | 40.2% | <.001 |
| Some College | 27.0% | 32.7% | .002 |
| College Graduate | 41.4% | 12.4% | <.001 |
| Married | 66.0% | 27.3% | <.001 |
| *Number of Prior Births* |  |  |  |
| 0 | 39.2% | 32.8% | .001 |
| 1 | 33.8% | 30.5% | .093 |
| 2 | 16.0% | 18.6% | .122 |
| 3+ | 10.9% | 18.1% | <.001 |
| *Body Mass Index* |  |  |  |
| Underweight | 3.0% | 4.7% | .052 |
| Normal Weight | 44.3% | 38.3% | .794 |
| Overweight | 24.8% | 25.3% | .047 |
| Obese | 27.9% | 31.7% | <.001 |
| *Household Income* |  |  |  |
| ≤ $16,000 | 16.9% | 44.6% | <.001 |
| $16,000, $40,000 | 22.5% | 30.2% | <.001 |
| $40,001 – $85,000 | 30.3% | 21.2% | <.001 |
| > $85,000 | 30.3% | 4.0% | <.001 |
| Number of Dependents | 2.94 (1.37) | 2.85 (1.43) | .129 |
| No Dental Insurance | 19.6% | 24.9% | <.001 |

*Abbreviations:* SD = Standard deviation
